# Environment-driven somatic mosaicism in brain disorders

**DOI:** 10.1186/s13073-016-0317-9

**Published:** 2016-05-23

**Authors:** Tracy A. Bedrosian, Sara Linker, Fred H. Gage

**Affiliations:** Laboratory of Genetics, The Salk Institute for Biological Studies, 10010 North Torrey Pines Road, La Jolla, CA 92037 USA

## Abstract

The identification of somatic mosaicism in the brain lends a new perspective to our understanding of the role of gene and environment interactions in psychiatric disease risk. Somatic mutations, such as retrotransposon insertions, that are precipitated by modern environmental factors may alter neuronal function and neurological traits, increasing the societal prevalence of mental disorders.

## Genomic diversity in human somatic neural cells

The presence of somatic cells that harbor distinct mutations within one individual is referred to as somatic mosaicism, and growing evidence suggests that this is both a feature of normal brains and, when unchecked, a hallmark of disease. Recent advances in single-cell sequencing technology have raised new opportunities to understand heterogeneity in the brain. New evidence challenges the basic assumption that neuronal genomes are static and identical among the cells of an individual. Instead, extensive genomic diversity has been identified among the neurons of individuals. A variety of mutations contribute to somatic mosaicism, including indels, copy number variants (CNVs), aneuploidy, retrotransposition, and single nucleotide polymorphisms (SNPs). Single-cell analyses recently revealed that a third of neural progenitors demonstrate aneuploidy, up to 40 % of neurons contain megabase-scale CNVs, and neurons contain de novo retrotransposon insertions. Although the exact rate of retrotransposition is unclear, estimates from single-cell analyses predict rates as low as <0.6 insertions per neuron and as high as 13 insertions per neuron, depending on the brain region and method of detection [1].

In contrast to germline mutations, which are inherited, somatic mutations are acquired and therefore affect only a subset of cells in the body, depending on when the mutation arises. A somatic mutation that occurs in an early progenitor during embryogenesis could be passed on to most cells of the brain and body. Alternatively, a somatic mutation that occurs in one neural progenitor in a neurogenic niche of the adult brain may only be transmitted to a small handful of cells. Depending on its genomic location, a mutation could have a range of effects on cellular function by altering gene expression or transcript splicing, affecting epigenetic modifications or enhancers, or generating novel fusion proteins, as in the case of long interspersed nuclear element (L1, also called LINE-1) retrotransposons [1, 2]. Considering the highly networked state of the brain, a small number of somatic mutations that affect cellular function could have far-reaching effects on neuronal circuitry.

**Fig. 1:**
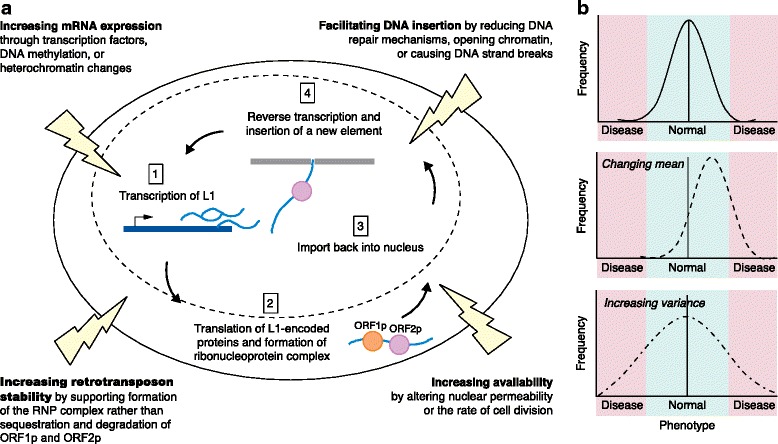
Environmental influence on retrotransposition and disease risk. **a** Environmental factors can increase the rate of retrotransposition by affecting cellular and molecular processes that act at different levels of the insertion cycle (*thunderbolts* represent environmental effects). (1) In the nucleus (*dotted line* represents the nuclear envelope), full-length long interspersed nuclear element (*L1*) retrotransposon elements are transcribed from an internal Pol II promoter. Environmental effects on transcription factor expression or binding, on DNA methylation of L1 elements, or on heterochromatin structure could increase the transcription of L1, introducing more substrate for potential retrotransposition. (2) L1 mRNA encodes two proteins: an RNA-binding protein (ORF1p) and a protein with endonuclease and reverse transcriptase domains (ORF2p). These proteins form a ribonucleoprotein (*RNP*) complex with L1 mRNA in cis-preference (that is, mRNA from the same L1 element), and this complex can be imported into the nucleus or sequestered into stress granules and degraded. Environmental factors may influence cellular defenses involved in the sequestration and degradation of L1 proteins, which could affect the amount of stable L1 RNP complexes available for import into the nucleus. (3) L1 RNP complexes are imported into the nucleus during cell division when the nuclear envelope is disrupted, or through an unknown import mechanism. Environmental effects on nuclear permeability or on the rate of cell division could increase the number of L1 RNP complexes imported into the nucleus, thus increasing availability of the machinery needed for retrotransposition. (4) Once inside the nucleus, L1 endonuclease nicks the genomic DNA at a TTAAA sequence and the L1 mRNA is inserted via target-primed reverse transcription (*pink circle* represents ORF2p reverse transcribing L1 mRNA). Environmental effects on DNA damage, DNA repair processes, or chromatin structure could affect the accessibility of the genomic DNA and its vulnerability to acquiring a new insertion. **b** Many neurological phenotypes, for example stress activity, are normally distributed, with extreme traits at the outer tails of the distribution predicting disease (*top graph*). The effect of high levels of somatic mutation on disease risk might be explained by models of additive or neutral risk. An extreme model would suggest that all somatic variation is associated with additive risk. In this case, the net result would be a shift in the mean of the respective phenotype (*middle graph*). Conversely, a more conservative model would posit that somatic events, much like germline mutations, have a largely neutral effect on the phenotype, with rare events having an equal chance of increasing or decreasing risk. In this case, an accumulation of normally distributed interacting effects would expand the variance of a distribution while keeping the mean constant (*bottom graph*). Environmental factors that accelerate the somatic mutation rate could ultimately expand the phenotypic diversity in an affected population beyond its natural state, potentially increasing disease risk in the population. If the phenotypic distribution were expanded or shifted, more individuals would exhibit extreme neurological traits at the outer tails of the distribution

Accumulating evidence suggests that environmental factors drive some types of somatic mutations, particularly retrotransposition and retrotransposon-associated events [1]. For example, environmental factors that increase L1 expression or impair DNA-damage responses may increase the rate of retrotransposition (Fig. 1a). L1 is the most abundant class of retrotransposon, comprising about 17 % of mammalian genomes. L1 generates new insertions by undergoing reverse transcription and inserting a copy of its own mRNA into the genome during cellular division, when the nuclear membrane breaks down to allow import of the L1 ribonucleoprotein complex. Most retrotransposon copies are truncated or otherwise mutated such that their mobilization is no longer possible, but 80–100 copies remain active in the common reference human genome. Although many cell types express retrotransposons, dividing cells such as neural progenitor cells may support increased levels of retrotransposition [1].

Somatic mosaicism resulting from retrotransposition or other types of mutations may represent a bridge between environmental and genetic factors that predispose individuals to brain disorders. A long-standing question has been how environmental experiences permanently alter the brain and contribute to disease risk later in life. Here, we explore the implications of somatic mosaicism in the brain generated in response to the environment and their consequences for mental health.

## Effects of genomic diversity and environmental factors on disease risk

The total disease risk for a given individual and disorder is an accumulation of risk from both inherited and non-inherited factors, particularly for certain mental disorders where environmental experience contributes to disease onset. A large proportion of total psychiatric disease risk is attributed to germline mutations, with heritability estimates ranging from 30 to 80 % [3]. Despite the strong inherited component, discordance between monozygotic twins demonstrates that a portion of psychiatric and other brain disease etiology is driven by non-inherited factors, such as environmental perturbations or somatic mutations. Exposure to environmental factors, such as urban living and early life trauma, confers non-inherited risk for some psychiatric disorders, such as schizophrenia [4]; but the mechanistic link between early environmental events and disease progression is still largely unknown. The delay between exposure and onset of disease suggests that the environment induces stable changes, such as somatic mutations, that have the potential to manifest later in life.

Traditionally, environmental effects have been examined in interaction with germline variants. Many of these inherited alleles are common in the general population, indicating that no genome is completely devoid of risk [5]. Instead, there is a landscape of polygenic risk, where each variant is associated with a small increase in the odds of developing a particular disorder. When accumulated, the set of variants, as a whole, can generate a disease-associated phenotype. Somatic mutations that are driven by environmental factors are layered onto this landscape of inherited risk and may contribute to psychiatric disease by causing ‘multiple hits’ in an individual who has an inherited genetic predisposition.

Although somatic retrotransposition occurs at low rates in the mammalian brain [1], we do not know the extent to which these variants affect disease risk. One possibility is that a disease state is associated with an increased load of new insertions that can drive transcriptional and functional diversity. Both cell lines from Rett syndrome patients and prefrontal cortex from schizophrenia patients exhibit an increased load of L1 insertions [1]. As mentioned earlier, depending on the stage of development in which these variants arise, the frequencies of cells that harbor the somatic allele can range from one single cell per individual to every cell in the body. Since somatic retrotransposition would affect only a portion of all of the neurons in an adult, it is possible that the functional consequences are present at the somatic level but that the phenotype is modulated at the level of the organism.

The true range of effects of somatic variants on neuropsychiatric phenotypes should become elucidated with additional research. Nevertheless, models of neutral risk or additive risk can be envisaged (Fig. 1b). The latter could result in an increase in phenotypic variance at the cellular level, which would be defined in the brain by the generation of a highly diverse set of neurons. Such diversity is indeed prevalent in the brain and is thought to be vital for complex information processing [1].

## Environmental effects on somatic diversity risk

Much of the evidence gathered to date for environmental effects on somatic diversity focuses on L1 retrotransposition. Environmental events drive somatic mosaicism by instigating molecular or hormonal events that create a permissive state in which genomic changes can occur. For example, exposing adult mice to voluntary exercise increases hippocampal neurogenesis, thus increasing the number of dividing and differentiating cells that are permissive to retrotransposition [6]. Similarly, steroid hormones activate L1 elements by binding an androgen response element in their promoter and increasing their expression [7]. Given the importance of diversification for adaptation, it seems reasonable that natural environmental factors drive genomic diversity to some extent as a form of plasticity.

Environmental factors that expand this diversity beyond natural variation may, however, cause genomic instability and increase disease risk. In modern societies, humans are exposed to an increasing array of pollutants and toxins, some of which are man-made and never before encountered in the environment. Synthetic hormones and organochloride pesticides increase the transcription of human L1 retrotransposons [7]. Exposure to heavy metals increases L1 retrotransposition rate three-fold, putatively by reducing DNA-repair processes that defend against insertions [8]. Benzo-a-pyrene, a pollutant resulting from residential wood burning, automobile exhausts, and cigarette smoke, both upregulates L1 expression and increases retrotransposition [9]. Even exposure to artificial light at night, which dysregulates circadian rhythms, increases retrotransposition through a mechanism related to depleted melatonin [10].

The incidence of mental disorders is said to have increased in recent decades, particularly in modernized societies, even accounting for improved detection and diagnoses. The trend may be related, in part, to high rates of somatic mutation driven by environmental factors. For example, prenatal immune activation in mice is associated with both increased rates of retrotransposition and behavioral deficits reminiscent of schizophrenia; in humans, a similar correlation exists between prenatal viral infection, schizophrenia, and L1 copy number [1]. An expanding list of brain disorders, including autism, Rett syndrome, Alzheimer’s disease, and drug addiction, is associated with increased somatic mutation rate. Interactions between the environment and somatic mutations in the brain may contribute to some of these neural disorders.

In summary, we suggest that somatic mutations may contribute to the onset of psychiatric or other brain disorders by interacting with inherited genetic variants and introducing permanent changes to neuronal function. Somatic mosaicism induced by differences in environmental experience may explain disease discordancy between monozygotic twins or behavioral variability among genetically homogeneous laboratory mice. Future studies should aim to elucidate a causal role of the environment in regulating somatic mutation rates and to clearly define its relationship with behavioral and neuronal phenotypes. With a deeper understanding of how environmental factors interact with somatic mutations in the human brain, it may become possible to better predict and prevent mental disorders in susceptible individuals.

## Abbreviations

CNV: copy number variant
L1: long interspersed nuclear element
SNP: single nucleotide polymorphism
